# Response to ‘Methodological flaws on “manual therapy for the pediatric population: a systematic review” by Prevost et al. (2019)’

**DOI:** 10.1186/s12906-020-03165-2

**Published:** 2021-01-05

**Authors:** Katherine A. Pohlman, Kristian Anderson, Beth Carleo, Brian Gleberzon

**Affiliations:** 1 Parker University, 2540 Walnut Hill Lane, Dallas, TX 75229 USA; 2 Performance Chiropractic, 4350 South Washington Street Suite 100, Grand Forks, ND 58201 USA; 3 Palmer College of Chiropractic, 4777 City Center Parkway, FL Port Orange, 32129 USA; 4 Canadian Memorial Chiropractic College, 6100 Leslie St., North York, ON M2H 3J1 Canada

**Keywords:** Pediatric, Manual therapy, Manipulation, Mobilization, Systematic review

## Abstract

Correspondence from Yu et al. identify methodological issues with the systematic review of manual therapy for pediatric manuscript. Like any research study, limitations are important for readers to keep in consideration when reviewing study findings. The primary authors maintain full confidence in the use of the review to provide practicing clinicians with a comprehensive overview of the limited and low-quality available evidence regarding manual therapies for the pediatric patient.

We first thank the authorship team for their careful critique. We do believe that additional insights into the purpose and context of this manuscript [1] will be helpful for readers, especially practicing clinicians, as the manuscript was designed specifically for them. With the ever-increasing quantity of literature, practicing clinicians are inundated with varied interpretations of this literature [2]. Our manuscript’s intent was to compile the currently available literature so that a comprehensive overview of the research would be available, and we maintain full confidence in that endeavor. However, we would like to acknowledgement the accuracy of the commentary’s concern for the manuscript being classified as a systematic review. While our study did use systematic review methodology, hindsight reveals that a better study designation for this manuscript is likely ‘a systematic search and review’ [3]; we are grateful to the authorship team for pointing this out.

A well-known concern for all systematic reviews is the use of inconsistent methodology [4]; that concern is not unique to this manuscript. Systematic reviews methodology includes a large number of decisions which impacts the results, which has a consequence of conflicting conclusions among systematic reviews on the same topic by different research groups [5]. Readers of systematic reviews must cautiously interpret the findings with respect to the review’s limitations and methodological decisions. Specifically, while our review’s search strategy was clearly described in the methods section, that strategy was also acknowledged as a limitation in the discussion section. With that in mind, selection bias is always a valid concern, even though the selection of the identified and included studies in our review was non-directional toward any particular outcome or finding, which stands as a marker for identifying biases (or lack thereof) in a study. Additionally, as identified in the commentary, we implemented cut-off points and summary assessments of the included studies’ results to easily describe synthesized outcomes. The development of these cut-offs and summary assessments were done a priori and were based on methodology conducted in other similar comprehensive review manuscripts [6, 7]. Ideally, the transparency of the syntheses described in the methods allows readers to disregard or modify as desired. The conclusion of the commentary states that this manuscript should not be used to inform the clinical management of pediatric patients. At no point in the original manuscript do the authors give clinical management advice [1]. In addition, the commentary’s authorship team provides no alternative source of information, leaving practicing clinicians with the same void that led to the development of the review. Without a resource, such as the original manuscript, the overwhelming process of identifying literature and assessing the quality of available research is likely to leave busy clinicians to base their clinical management options on biases of clinical experience [2]. The original manuscript was to be used as a starting point for clinicians to discover what research studies are available (or not available) for a given condition. When further information is needed, a clinician could also use the review to begin their evaluation of the referenced manuscripts and any more recent publications. This approach combined with a clinicians’ experience and, perhaps most importantly, the patient’s (or in the case of children, their decision maker’s) preferences and values enables practitioners to approach patient care from an evidence-based perspective [8].

Constructive critiques and transparent professional disagreements in the interpretation of data findings allow for research in the field to advance. In this spirit, we hope that readers take the concerns outlined in the respective commentary seriously. Altogether, this series of manuscripts is a noteworthy example of the need for more collaborative research to bridge clinician-focused investigative teams to conduct useful, rigorous, and comprehensive studies. As a community of scientists, we must all continue to strive to aid each other in the quest for knowledge to provide clinicians with the tools they need to provide optimal patient care.

## Data Availability

Not Applicable.
